# The clinical value of serum-related indexes in differentiating simple premature thelarche from idiopathic central precocious puberty

**DOI:** 10.12669/pjms.40.3.7447

**Published:** 2024

**Authors:** Hai-jing Cui, Peng Liu, Xin-guang Liu, Wen-zhong Du, Xia Wang

**Affiliations:** 1 Hai-jing Cui, Department of Children’s Health, Children’s Hospital of Hebei Province, Shijiazhuang 050011, Hebei, P.R. China; 2 Peng Liu, Department of Medical Insurance, Children’s Hospital of Hebei Province, Shijiazhuang 050011, Hebei, P.R. China; 3 Xin-guang Liu, Department of Medical Laboratory, Children’s Hospital of Hebei Province, Shijiazhuang 050011, Hebei, P.R. China; 4 Wen-zhong Du, Department of Quality Control, Children’s Hospital of Hebei Province, Shijiazhuang 050011, Hebei, P.R. China; 5 Xia Wang, Department of Children’s Health, Children’s Hospital of Hebei Province, Shijiazhuang 050011, Hebei, P.R. China

**Keywords:** Simple premature thelarche, Idiopathic central precocious puberty, Vitamin-D, Sex hormones, Insulin-like growth factor, Leptin

## Abstract

**Objective:**

To explore the changes of serum-related indexes at different time points, so as to identify the critical time of converting from simple premature thelarche (PT) to idiopathic central precocious puberty (ICPP).

**Methods:**

This is a retrospective study. The subjects of the study were 50 girls with PT who were admitted to the Children’s Hospital of Hebei Province from January 2019 to September 2020. The enrolled 50 children were divided into the conversion group(n=12) and the non-conversion group(n=38) according to whether PT was converted into ICPP during follow-up. Furthermore, the levels of serum-related indexes and uterine and ovarian volumes were compared after the diagnosis of PT.

**Results:**

The IGF-1 and IGFBP-3 levels of children in the conversion group began to change significantly from six months after the diagnosis, with statistically significant differences when compared with the levels of children at the initial diagnosis, three months and those of the non-conversion group at the same time points (*p<*0.05). The levels of vitamin-D, DHEA and leptin began to change significantly at nine months after the diagnosis (*p<*0.05). Besides, uterine and ovarian volumes in the conversion group began to increase significantly six months after the diagnosis, with statistically significant differences when compared with those in the non-conversion group (*p<*0.05).

**Conclusion:**

Findings in our study suggest that regular monitoring of vitamin-D, IGF-1, IGFBP-3, DHEA and leptin levels, and uterine and ovarian volumes can predict the conversion from PT to ICPP at an early stage.

## INTRODUCTION

Precocious puberty is defined as the presence of secondary sexual characteristics in girls under the age of eight or boys under nine years old.^1^ Due to the impact of diet and environmental factors, as well as the improvement of family living conditions, there is an accelerated trend of the growth and development of children around the world, accompanied by an obviously increased incidence of precocious puberty (incidence ratio of boys: girls, about 1:5).^2^,^3^ Precocious puberty may produce a negative effect on children’s psychological and physical health (e.g., height growth). Moreover, precocious puberty in girls can increase the risk of diabetes, hypertension and cardiovascular disease in adulthood.^4^,^5^ According to the pathogenesis, precocious puberty can be divided into true precocious puberty [idiopathic central precocious puberty (ICPP) mostly clinically], partial precocious puberty [simple premature thelarche (PT) mostly clinically] and pseudo-precocious puberty [also known as peripheral precocious puberty(PPP)], in which ICPP accounts for >92% of children with precocious puberty.^6^

Meanwhile, PT is also the most common type of precocious puberty. PT is a non-self-limited disease, some of which can disappear voluntarily, and some can be converted into ICPP. In terms of the risk factors reported by previous studies,^7^,^8^ PT in girls are closely related to hypocorticism, hypothalamic-pituitary-gonadal axis dysfunction, and genetic and environmental factors. Meanwhile, the development of ICPP shows an intimate association with the levels of vitamin-D, sex hormones, insulin-like growth factor-1 (IGF-1), leptin, etc.^9^ Gonadotropin-releasing hormone (GnRH) stimulation test has been accepted to be the gold standard for diagnosing ICPP so far,^10^ which is also extensively employed to assist in the differential diagnosis of precocious puberty in children. In this study, the time point of conversion from PT to ICPP was discussed by monitoring the levels of vitamin-D, sex hormone, IGF-1 and leptin, as well as uterine and ovarian volumes in children. It is expected to detect and prevent the conversion of PT into ICPP at an early stage, so as to reduce the incidence of ICPP, ensure children’s physical and mental health, and promote their normal growth and development.

## METHODS

This is a retrospective study. The subjects of the study were 50 girls with PT who were admitted to the Children’s Hospital of Hebei Province from January 2019 to September 2020. The time interval for further consultation and examination in the hospital was every three months. Children were followed up until December 2021, without withdrawal during the follow-up period. The enrolled 50 children were divided into the conversion group (n=12) and the non-conversion group (n=38) according to whether PT was converted into ICPP during follow-up. General data of all children were collected at the time of initial diagnosis, including age, body mass index (BMI), breast Tanner stage, uterine and ovarian volumes, etc.

### Ethical Approval:

The study was approved by the Institutional Ethics Committee of Children’s Hospital of Hebei Province (No.:2032305; date: February 08, 2023), and written informed consent was obtained from all children’s families.

### Inclusion criteria:

- Children who met the diagnostic criteria of PT: unilateral or bilateral breast enlargement with normal gonads on imaging, no secondary sexual signs, and age<eight years old; and
- Children without a history of taking estrogen-containing food by mistake.

### Exclusion criteria:

- Children with breast development caused by other primary diseases;
- Children with contraindication of imaging examination;
- Children with long-term medication history.

Sampling and storage: An amount of five ml of fasting venous blood was collected from each child at the time of initial diagnosis, as well as at three, six, nine and 12 months after diagnosis of PT. The blood samples were then subjected to centrifugation at 2500 r/min for 10 min, with a centrifugal radius of 15 cm. The obtained supernatant was collected and stored at -80°C for further use. Vitamin-D was determined by using high-performance liquid chromatography.

The levels of IGF-1 and insulin growth factor binding protein-3 (IGFBP-3) were detected by the chemiluminescence method. Radioimmunoassay was performed to detect dehydroepiandrosterone (DHEA), and an enzyme-linked immunosorbent assay was employed to detect leptin. Uterine and ovarian volumes (Volume=long diameter × anteroposterior diameter × transverse diameter×0.5233) were recorded by ultrasonic examination to evaluate the development of the uterus and ovary. Children were followed up for at least 12 months via outpatient visits.

### Statistical analysis:

The SPSS26.0 software was used for statistical analysis in this study. The measurement data were expressed by mean ± standard deviation (*χ̅*±*S*), and the counting data were presented in n (%), with *t* test and c² test adopted for inter-group and inter-group comparison, respectively. Meanwhile, Pearson correlation analysis was used to examine the correlation between variables, and linear regression was utilized to perform regression analysis. The power of test / confidence interval is 95%. The difference was statistically significant when *p<*0.05.

## RESULTS

There was no significant difference in age, BMI, Tanner stage of breast and ovarian volume between the two groups (*p>*0.05), with comparability between groups. (Table-I). There was no significant change in vitamin-D, IGF-1, IGFBP-3, DHEA and leptin levels of children in the non-conversion group in different time periods, without a statistically significant difference. (*p>*0.05) (Table-II).

**Table-I:** Comparison of general data between the two groups.

Pathogen	Non-conversion group (n=38)	Conversion group (n=12)	t/χ^2^ value	P value
Age (years)	6.66±0.78	6.75±1.06	0.327	0.745
BMI (kg/m^2^)	16.13±1.91	16.62±1.20	0.823	0.414
Tanner stage			0.049	0.825
Stage-B II	24	8		
Stage-B III	14	4		
Uterine volume	1.80±0.29	1.87±0.29	0.670	0.506
volume	1.76±0.27	1.89±0.23	1.504	0.139

**Table-II:** Comparison of serum-related indexes between the two groups of children in different time periods (*χ̅*±*S*).

Groups	Time	Vitamin-D (nmol/L)	IGF-1 (ng/mL)	IGFBP-3 (μg/mL)	DHEA (μg/L)	Leptin (ng/mL)
Non-conversion group	Initial diagnosis	84.24±7.69	129.71±8.87	3.69±0.37	41.41±5.66	7.52±2.47
	3 months	84.09±7.66	131.67±8.83	3.66±0.27	42.52±5.48	7.73±2.15
	6 months	83.85±7.56	131.72±6.95	3.67±036	42.78±6.89	7.99±2.19
	9 months	82.01±7.04	132.81±8.87	3.64±0.38	43.24±6.74	8.01±1.84
	12 months	80.91±6.95	131.65±7.03	3.66±0.34	41.32±6.96	7.93±2.03
Conversion group	Initial diagnosis	84.02±7.15	131.32±7.15	3.80±0.30	42.83±5.92	7.32±2.65
	3 months	81.88±7.08	132.36±6.33	3.81±0.31	43.87±5.57	7.98±1.99
	6 months	79.22±6.98	162.41±9.01	4.38±0.34	43.45±5.16	8.83±1.93
	9 months	72.17±7.07	183.62±7.15	5.23±0.30	57.75±4.26	14.13±1.92
	12 months	64.78±6.12	222.59±6.94	5.69±0.25	64.54±6.58	18.98±3.12

At the time of initial diagnosis and at three months, no significant difference was found in uterine and ovarian volumes between the two groups (*p>*0.05). Since the six months, uterine and ovarian volumes of children at nine and 12 months increased in both groups when compared with those of the previous time periods, especially significant in the conversion group, with statistically significant differences in intra-group comparison and inter-group comparison with the non-conversion group (*p<*0.05) (Table-III).

**Table-III:** Comparison of the uterus and ovary development in two groups of children in different time periods (*χ̅*±*S*).

Items	Non-conversion group					Conversion group				

	Initial diagnosis	3 months	6 months	9 months	12 months	Initial diagnosis	3 months	6 months	9 months	12 months
Uterine volume (ml)	1.82±0.28	1.87±0.27	1.96±0.27	1.97±0.27	1.98±0.28	1.87±0.29	1.92±0.29	2.68±0.30	2.80±0.29	2.98±0.29
Ovarian volume (ml)	1.76±0.27	1.81±0.28	2.27±0.26	2.35±0.24	2.45±0.26	1.89±0.23	1.91±0.24	2.47±0.26	2.70±0.21	3.01±0.23

**Fig.1:**
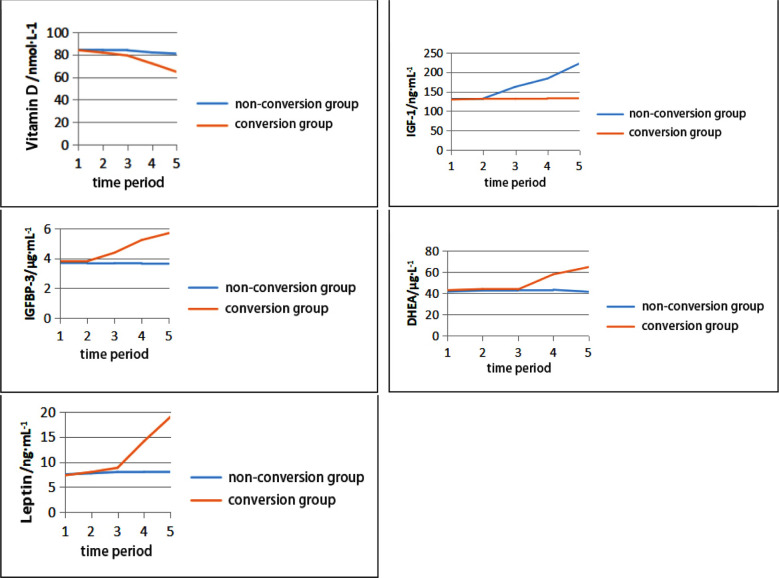
Line chart of variation of different serum-related indexes with time. ***Note:*** 1: initial diagnosis, 2: 3 months, 3: 6 months, 4:9 months, and 5: 12 months.

All serum-related indexes fluctuated with time, and there existed differences at different time periods. The proposed fluctuation might have a certain impact on the occurrence of ICPP. In the conversion group, the levels of vitamin-D, IGF-1 and IGFBP-3 began to change significantly at six months, while the levels of DHEA and leptin changed most significantly at nine months. There were statistically significant differences when compared with the non-conversion group in the same periods (*p<*0.05), as presented in (Fig.1).

**Fig.2:**
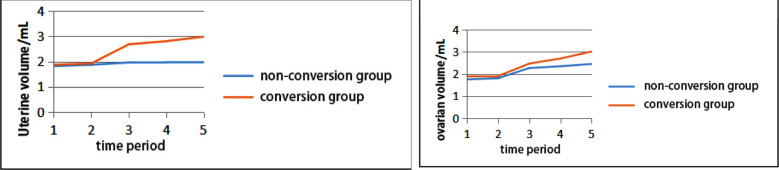
Line chart of uterine and ovarian volume fluctuation with time. ***Note:*** 1, initial diagnosis, 2, 3 months, 3, 6 months, 4, 9 months, and 5, 12 months.

Uterine and ovarian volumes fluctuated with time, with differences at different time periods, which might have a certain impact on the occurrence of ICPP. As shown in (Fig.2), uterine and ovarian volumes in the conversion group began to change significantly at six months, which were significantly different from those in the non-conversion group at the same time periods. Statistically significant differences were detected when compared with the non-conversion group in the same periods (*p<*0.05).

## DISCUSSION

In our study, the vitamin-D level began to decrease half a year after the identification of breast development in children from the conversion group, which was significantly lower than the normal level (*p<*0.05), which was the same as the results of several clinical studies. Furthermore, IGF-1 and IGFBP-3 levels in children with precocious puberty were significantly higher than those in normal children of the same age and sex. The reasons could be the hypothalamus-pituitary-gonad axis is initiated to activate the growth axis, thus stimulating the increased production of IGF-1 and IGFBP-3 in children with precocious puberty. The increased IGF-1 and IGFBP-3 levels can play a negative feedback-regulating role in the activities of the growth axis. Moreover, IGF-1 and IGFBP-3 can regulate the release of GHRH of the hypothalamus-pituitary, resulting in the early growth and development of children finally.^11^,^12^

In this study, IGF-1 and IGFBP-3 levels in children from the conversion group began to increase significantly at six months after the confirmation of breast development, both of which were higher than those in the non-conversion group (*p<*0.05). These trends were consistent with the theory mentioned above, indicating that changes in IGF-1 and IGFBP-3 levels can be indicators of precocious puberty in children. Simultaneously, DHEA is the major indicator of the activity of the adrenal gland and the level of adrenal androgen. The androgen secreted by the adrenal gland is the main source of female androgens, and some androgens are secreted by the ovary.^13^ The increase in female adrenal androgen level will cause breast development and gonadal maturation, shorten the duration of the growth spurt in children, and lead to a shorter height than normal children. At the same time, leptin is mainly produced in white adipose tissue.

The levels of leptin have been demonstrated to be strongly associated with obesity, sexual development and reproductive function, as well as the time of sexual maturity; and leptin can eliminate the inhibitory effect of neuropeptide Y on hypothalamic GnRH.^14^ It can regulate puberty onset and reproductive function through the hypothalamus-pituitary-gonad axis, promote the pituitary secretion of luteinizing hormone and follicle-stimulating hormone, and finally promote GnRH release, leading to early puberty initiation. Consistent with multiple clinical studies, in our study, the levels of DHEA and leptin in children with ICPP were highly increased at nine months after breast development, which were higher than those in normal children (*p<*0.05). Collectively, results in our study suggest the emergence of abnormal serological indicators about six months after early breast development, showing deterioration over time.

So far, there is a poor understanding in the definite mechanism related to early breast and ovarian development in young children. According to previous research perspective, patients with early breast development after the GnRH stimulation test showed follicle-stimulating hormone secretion-dominant, which was significantly higher than that of normal controls.^15^ Meanwhile, the increase of ovarian microcapsules was considered to increase the sex hormone precursors secreted by estrogen and the adrenal gland, which was also an incentive for early breast development.^16^ Moreover, early follicular development can also stimulate early breast development.

Precocious puberty is a common disorder of puberty development in children. It is known to be a multifactorial disease, among which nutritional status is an important factor affecting sexual development.^17^ The majority of children with ICPP have over-nutrition owing to long-term consumption of fried food, beverages, hormone-containing health products and supplements.^18^ Meanwhile, environmental factors are also the principal reasons for ICPP. Long-term exposure to plasticizing agents and detergents will lead to sexual development at the younger age.^19^ In addition, other risk factors also include premature exposure to sexual information, long hours of illumination and sleep with lights on, which may induce the secretion of pituitary gonadotropins.^20^ ICPP and PT are the most common types among various subtypes of precocious puberty. The differentiation between ICPP and PT has been a hot topic in clinical practice.

Similar to estrogen, vitamin-D is also a steroid hormone that may participate in the development and maturation of the female reproductive system. Moreover, the level of vitamin-D has been reported to be associated with precocious puberty in girls. For example, some researchers proposed that the age of menarche in girls was related to the level of vitamin-D, and girls would experience a younger age of menarche when there was a lower level of vitamin-D in serum.^21^

According to the results of our study, in children with ICPP, uterine and ovarian volumes were increased gradually from six months after early breast development, both of which were larger than those of children with PT, with a statistically significant difference (*p<*0.05). This result indicates a positive correlation between increased ovarian volume with early breast development of children, which is consistent with the above point of view.

### Limitation:

It includes a small sample size, short period of follow-up, etc., which may produce a certain impact on the level of evidence of this study. Findings in this study are expected to be confirmed through further research based on a long-term follow-up with larger sample size.

## CONCLUSIONS

To sum up, serum vitamin-D, IGF-1, IGFBP-3, DHEA and leptin levels, and uterine and ovarian volumes may change significantly 6-9 months after the diagnosis of PT, which can be used as the effective indexes to differentiate PT and ICPP. Findings in our study suggest that evaluation of treatment effect at an early stage can be benefited from active treatment after the discovery of early breast development, as well as regular monitoring of vitamin-D, IGF-1, IGFBP-3, DHEA and leptin levels.

### Authors’ Contributions:

**HC** and **PL:** Carried out the studies, participated in collecting data, and drafted the manuscript, and are responsible and accountable for the accuracy and integrity of the work.

**XL** and **WD:** Performed the statistical analysis and participated in its design.

**XW:** Participated in acquisition, analysis, or interpretation of data and draft the manuscript. All authors read and approved the final manuscript.
